# Cloning, characterization, and spatio-temporal expression patterns of *HdhSPARC* and its responses to multiple stressors

**DOI:** 10.1038/s41598-024-51950-7

**Published:** 2024-01-26

**Authors:** Md Abu Hanif, Shaharior Hossen, Cheol Young Choi, Kang Hee Kho

**Affiliations:** 1 Department of Fisheries Science, Chonnam National University, Yeosu, 59626 South Korea; 2 Division of Marine BioScience, National Korea Maritime and Ocean University, Busan, 49112 South Korea

**Keywords:** Genetics, Gene expression

## Abstract

SPARC is an extracellular Ca^2+^-binding, secreted glycoprotein that plays a dynamic role in the growth and development of organisms. This study aimed to describe the isolation, characterization, and expression analysis of *HdhSPARC* in Pacific abalone (*Haliotis discus hannai*) to infer its potential functional role. The isolated *HdhSPARC* was 1633 bp long, encoding a polypeptide of 284 amino acid residues. Structurally, the SPARC protein in abalone is comprised of three biological domains. However, the structure of this protein varied between vertebrates and invertebrates, as suggested by their distinct clustering patterns in phylogenetic analysis. In early development, *HdhSPARC* was variably expressed, and higher expression was found in veliger larvae. Moreover, *HdhSPARC* was highly expressed in juvenile abalone with rapid growth compared to their slower-growing counterparts. Among the testicular development stages, the growth stage exhibited higher *HdhSPARC* expression. *HdhSPARC* was also upregulated during muscle remodeling and shell biomineralization, as well as in response to different stressors such as heat shock, LPS, and H_2_O_2_ exposure. However, this gene was downregulated in Cd-exposed abalone. The present study first comprehensively characterized the *HdhSPARC* gene, and its spatio-temporal expressions were analyzed along with its responses to various stressors.

## Introduction

Aquaculture is one of the world's fastest-growing economic sectors^1^. Abalone are commercially important marine gastropod mollusks, that are widely distributed along tropical and temperate coastal regions^2^. Abalone is often referred to as the ‘ginseng of the ocean’ due to its health-promoting effects owing to its nutritional composition and bioactive molecules^3^. Currently, abalone production mainly relies on aquaculture, with fishery yields contributing very little to meet the demand for this resource. Abalone aquaculture completely depends on hatchery-produced seed^4^. There are thousands of genes and enzymes regulating the growth and development of abalone through different molecular mechanisms.

The extracellular matrix (ECM) acts as a reservoir for a complex combination of cytokines and growth factors with diverse structural and regulatory functions^5^. ECM proteins are known to be involved in a variety of activities, including muscle cell development, structure maintenance, and tissue repair, through the modulation of growth factors and cell–matrix signal transduction pathways^6^. SPARC (secreted protein acidic and rich in cysteine) is a Ca^2+^-binding glycoprotein found in extracellular matrix^7^ secreted by different cell types and plays a pleiotropic role^8^ throughout the different developmental stages of vertebrate and invertebrate species. This multifunctional secreted protein has a high affinity for cations, provides support to the extracellular matrix proteins, and mediates a wide range of growth factors and cytokines^9^ activities, including embryogenesis^10^, shell biomineralization^11^, muscle tissue regeneration^12^, and stress responses^13^, via cell–matrix interaction. SPARC deficiency in mice was previously reported to reduce glucose tolerance and insulin secretion^14^, which may influence the growth and development of aquaculture organisms. In contrast, overexpression of SPARC increases muscle strength, muscle mass, glucose transporter activity, and oxidative phosphorylation in mitochondria^15^. Inactivation of SPARC is also reported to impair gonad functions^16^. Moreover, although the exact function of SPARC in the early development (embryogenesis) of animals remains largely unknown, some studies have reported its involvement in embryo viability, tissue morphogenesis, and hematopoiesis^19^.

Tissue regeneration is a vital physiological development process that involves the growth and renewal of various cells and tissues to overcome biological disturbance and adaptation^15^. SPARC is a novel myokine^18^ secreted from the myocytes of muscle tissue. The SPARC molecule is known to have regenerative effects such as the regeneration of muscle tissue after physical or pathological damage through repair, cellular differentiation, and remodeling mechanisms^16^. In teleosts, SPARC is expressed in skeletal muscle during regeneration and development, as well as in satellite cells, suggesting that SPARC has a vital role in the skeletal muscle compartment^19^.

The shell of mollusks is an intricate natural structure that forms through a complex and highly controlled calcium metabolism process known as biomineralization^20^. In mollusks, several shell matrix proteins, including SPARC, have been isolated in the extrapallial fluid, which contains a variety of ions (Ca^2+^, Mg^2+^, K^+^, Na^+^, and HCO_3_^–^) that bind to CaCO_3_ crystals^11^. Previous studies have characterized the role of SPARC in shell formation in different mollusk species, including the Pacific oyster^21^, the pearl oyster^22^, and the sea snail^23^. A wide range of functional elements such as bone, shell, teeth, and coral reefs are the products of biomineralization process^24^.

In aquaculture systems and natural habitats, a variety of stressors including anthropogenic activities, pathogens, and climate change can affect vital physiological functions such as the growth and development of aquatic animals. Hydrogen peroxide (H_2_O_2_)^25^, cadmium (Cd)^26^, lipopolysaccharide (LPS)^27^, and heat stress^20^ are among the most common stressors that can alter SPARC expression and its related functional activity in aquaculture animals.

This study aimed to molecularly characterize the multifunctional matricellular glycoprotein SPARC in Pacific abalone (*H. discus hannai*) and investigate its transcriptional regulation during embryogenesis, postnatal development, remodeling and repair, and immune response through expression analysis. Regulation of SPARC in farmed abalone may contribute to the abalone aquaculture industry and related future research.

## Results

### HdhSPARC sequence analysis

The cDNA sequence encoding *H. discus hannai* SPARC was cloned from the mantle tissue and designated as *HdhSPARC*. The full-length sequence of *HdhSPARC* cDNA (GenBank Accession No. OM937904.1) was 1633 bp long, including a poly-A tail (Fig. S1), and its 5′- and 3′-untranslated regions (UTR) were 240 bp and 538 bp long, respectively. A putative polyadenylation signal (AATAAA) was identified in its nucleotide sequence 21 bp upstream of the poly-A tail. The ORF of the *HdhSPARC* cDNA sequence was 855 bp with 284 deduced amino acids.

The motif scan analyses detected a single amidation site X-G-[R/K]-[R/K] at the amino acid position 125–128 (GGRR) in the C-terminal region. A total of 18 phosphorylation sites were observed, of which seven were protein kinase C (PKC) phosphorylation sites, [S/T]-X-[R/K], at positions 107–109 (STK), 221–223 (SRR), 231–233 (SLK), 250–252 (SNR), 267–269 (SKK), and 271–273 (SDK); eleven were casein kinase II phosphorylation sites, [S/T]-X(2)-[D/E], 115–118 (SECE), 180–183 (SRDE), 214–217 (SPQD), 221–224 (SRRE), 244–247 (SMCD), and 255–258 (TLTD); three were cAMP- and cGMP-dependent protein kinase phosphorylation sites, [R/K](2)-X-[S/T], at position 150–153 (RKLT), 252–255 (RKIT), and 268–271 (KKIS); two N-myristoylation sites at position 132–137 (GCSNAR) and 260–265 (GACLGV); and two N-glycosylation sites at position 110–113 (NQTY) and 199–202 (NHTE) (Fig. S1).

**Figure 1:**
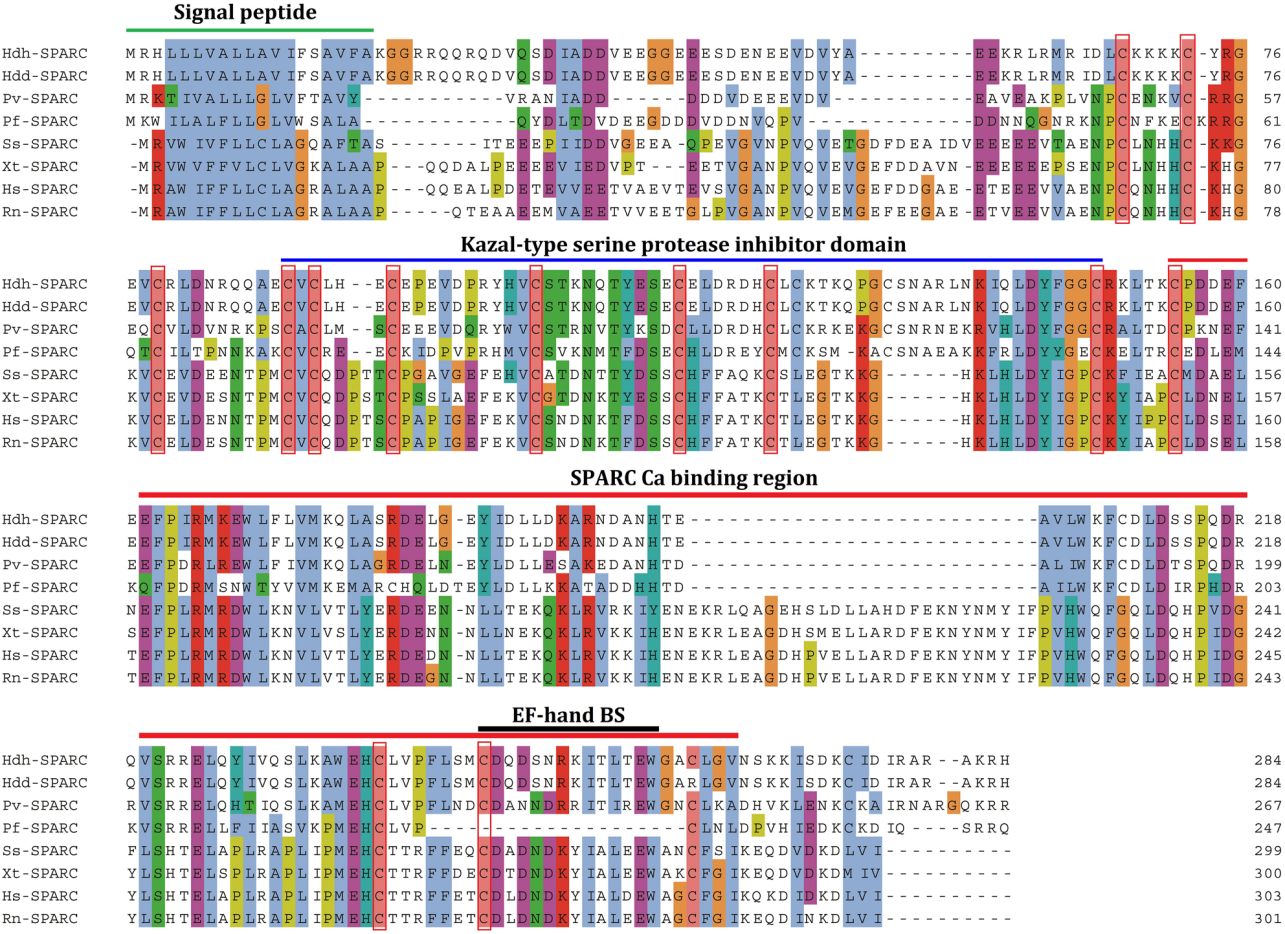
Multiple sequence alignment of HdhSPARC from deduced amino acid sequences of *H. discus hannai* (OM937904.1), *H. discus discus* (BAK22657.1), *Pinctada fucata* (AND99565.1) *Patella vulgata* (CCJ09602.1), *Salmo salar* (ACH70833.1), *Xenopus tropicalis* (AAT01218.1), *Rattus norvegicus* (NP_036788.1), and *Homo sapiens* (AAA60570.1). The blue letters indicate hydrophobic residues, red letters signify positively charged residues, magenta letters denote negatively charged residues, green letters represent polar residues, pink letters are for cysteine residues, orange letters for glycine residues, yellow letters for proline residues, cyan letters indicate aromatic residues, and white letters are for the unconserved residues. The green line indicates the signal peptide, and the red boxes indicate the conserved cysteine residues. The red line indicates the SPARC domain.

In the multiple sequence alignment, 14 conserved cysteine residues were found to be conserved when aligned with SPARC of *H. discus hannai*, *H. discus discus*, *Pinctada fucata*, *Patella vulgata*, *Salmo salar*, *Xenopus tropicalis*, and *Homo sapiens* (Fig. 1). The first 19 residues were detected as the signal peptide for this secreted protein. The SPARC domain was found in the C-terminal region of the sequence. Moreover, the N-terminal region of the HdhSPARC was more conserved than the C-terminal region for vertebrates and invertebrates. Among the species 13 cysteine residues were found conserved.

### Structure of the HdhSPARC protein

**Figure 2:**
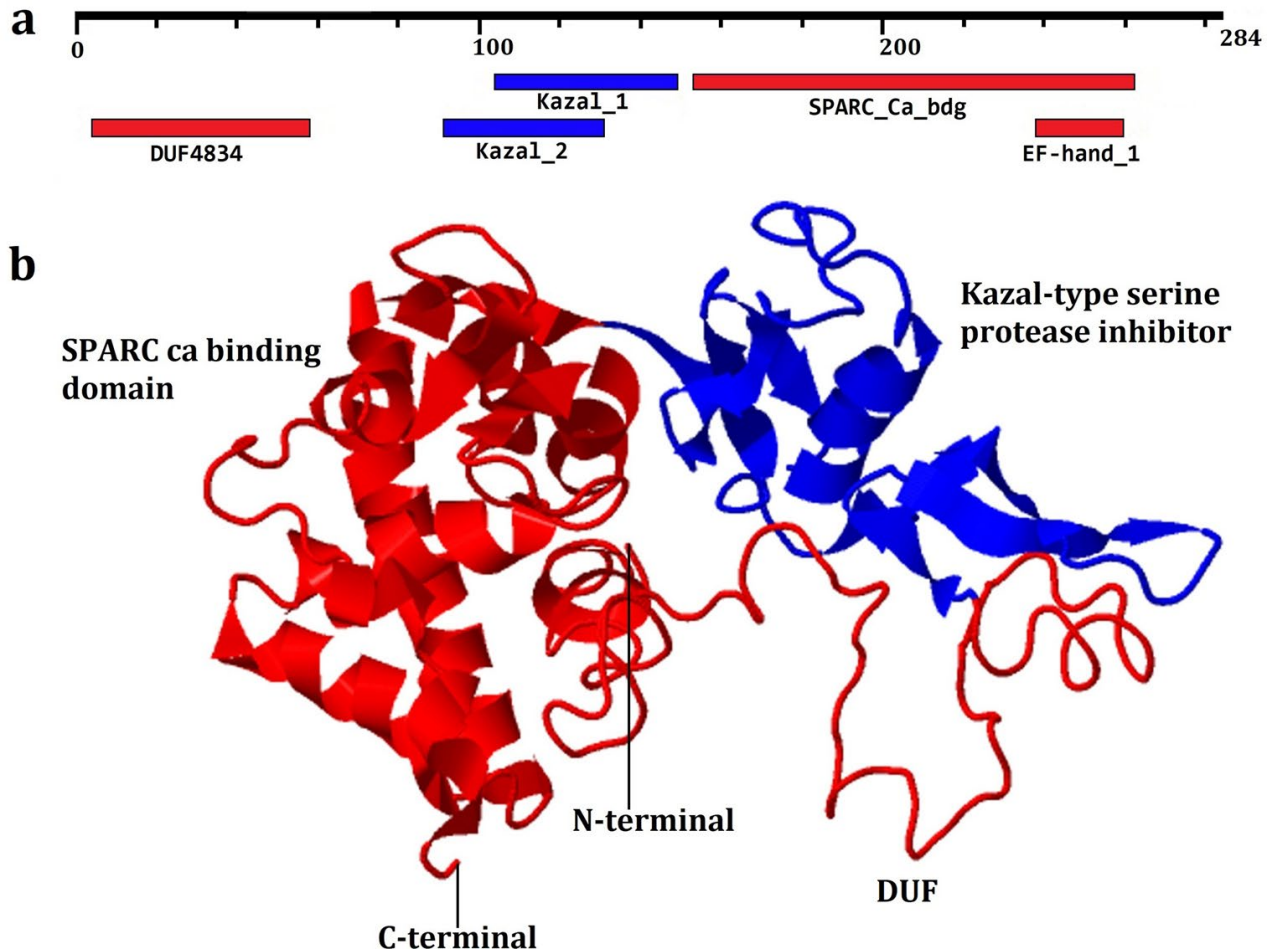
Structure of the HdhSPARC protein. (**A**) three functional domains detected in HdhSPARC, (**B**) three-dimensional structure with the conserved domain.

Structurally, HdhSPARC exhibits three concordant functional domains, including the DUF domain (N-terminal region), the Kazal-type serine protease inhibitor domain, and the SPARC Ca-binding domain with EF-hand motif (Fig. 2a). The constructed three-dimensional structure was dominated by coils and helixes, with very few strands. The helix and strands were mostly found in the C-terminal region (Fig. 2b).

### Phylogenetic analysis

**Figure 3:**
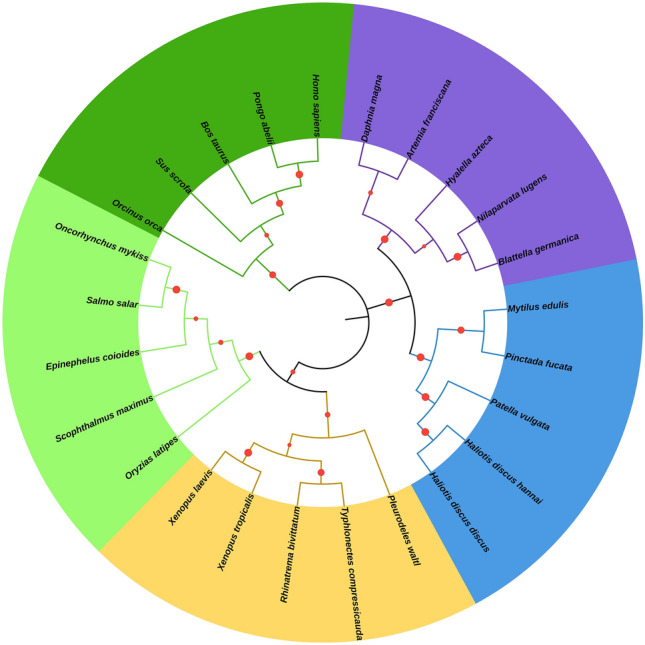
Phylogenetic tree of SPARC constructed via the maximum likelihood method after ClustalW alignment based on amino acid residues of different vertebrate and invertebrate species. The red circle in the tree indicate bootstrap probability. The green cluster indicates Mammalia, yellow for Amphibia, light green for Actinopterygii, blue for Gastropoda, and violet for Branchiopoda. The following protein sequences with their accession id were used to construct the phylogenetic tree: *Homo sapiens* (AAA60570.1), *Pongo abelii* (NP_001127042.1), *Bos taurus* (ABQ12988.1), *Sus scrofa* (AAX83050.1), *Orcinus orca* (XP_004280373.1), *Pleurodeles waltl* (AWQ28573.1), *Rhinatrema bivittatum* (AWQ28578.1), *Typhlonectes compressicauda* (AWQ28577.1), *Xenopus laevis* (CAA44350.1), *Xenopus tropicalis* (AAT01218.1), *Oryzias latipes* (AAT01217.1), *Scophthalmus maximus* (AGW25370.1), *Epinephelus coioides* (ACJ66296.1), *Oncorhynchus mykiss* (AAC99813.1), *Salmo salar* (ACH70833.1), *H. discus discus* (BAK22657.1), *H. discus hannai* (OM937904.1), *Patella vulgata* (CCJ09602.1), *Pinctada fucata* (BAK22656.1), *Mytilus edulis* (CAG2244558.1), *Hyalella azteca* (XP_018022874.1), *Artemia franciscana* (BAB20042.1), *Daphnia magna* (XP_045029097.1), *Blattella germanica* (CZQ50751.1), and *Nilaparvata lugens* (UPH52990.1).

The constructed phylogenetic tree based on the amino acid sequence using the maximum likelihood method formed five clusters (Mammalia, Amphibia, Actinopterygii, Gastropoda, and Branchiopoda), of which three were vertebrate species and two were invertebrate species. Among the two invertebrate clusters, the HdhSPARC clade with Hdd-SPARC and Pv-SPARC of gastropod clustered with high bootstrap value, whereas the branchiopod protein formed a separate cluster (Fig. 3). Phylogenetically, the invertebrate species were closer than the vertebrate species.

### Tissue-specific mRNA expression of *HdhSPARC*

Among the different tissues of Pacific abalone, *HdhSPARC* mRNA was most significantly (*p* < 0.05) expressed in the testis, followed by the mantle, muscle, and digestive gland tissue (Fig. S2). The lowest expression was observed in the ovary tissue. These findings were consistent with those of our semi-quantitative RT-PCR expression analysis (Fig. S3).

### *HdhSPARC* expression in embryonic and larval development of Pacific abalones

**Figure 4:**
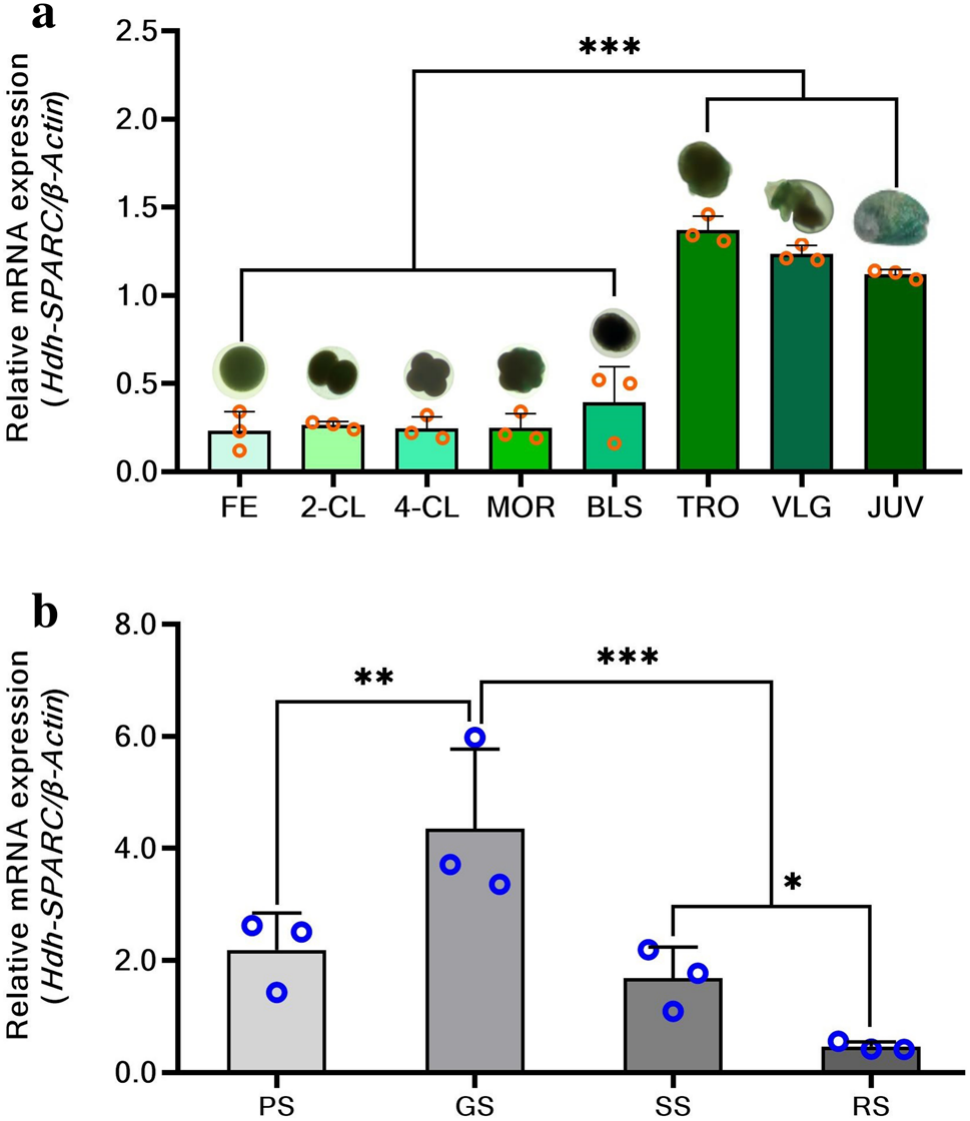
Development stage specific mRNA expression of *HdhSPARC* in Pacific abalone. (**a**) embryonic and larval developmental (FE, fertilized egg; 2-CL, 2-cell, 4CL, 4-cell, MOR, morula, BLS, blastula, TRO, trochophore; VLG, veliger; JUV, juvenile), (**b**) testicular development (PS, proliferative stage; GS, growth stage; SS, spawning stage; RS, resting stage). (^ns^*p* > 0.05; *p < 0.05; ***p < 0.001).

*HdhSPARC* mRNA was dynamically expressed during the embryonic and larval development stages. After fertilization, *HdhSPARC* mRNA was minimally expressed until the blastula stage, and no significant differences were observed. However, after the blastula stage, the expression increased significantly (*p* < 0.05) in the trochophore stage, followed by the veliger and juvenile stages (Fig. 4a).

*HdhSPARC* mRNA was differentially expressed in testis tissue throughout the different development stages examined herein. The growth stage showed significantly higher (*p* < 0.05) expression. *HdhSPARC* expression decreased thereafter, with the lowest expression being observed in the resting stage (Fig. 4b).

### *HdhSPARC* expression in Pacific abalones exhibiting different growth patterns

**Figure 5:**
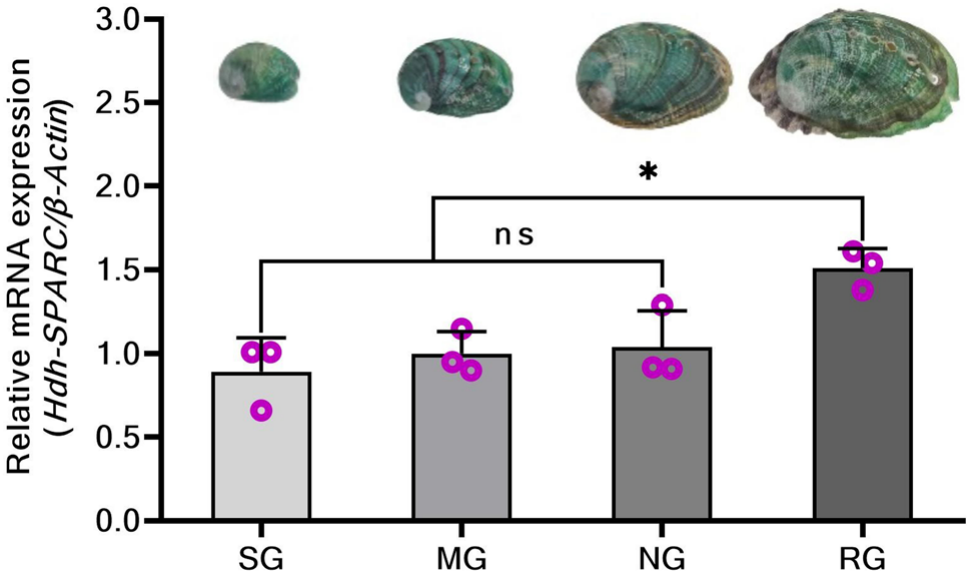
Expression of *HdhSPARC* in mantle and muscle tissue of Pacific abalones exhibiting different growth patterns. SG, stunted growth; MG, minimum growth; NG, normal (average) growth; RG, rapid growth. (^ns^*p* > 0.05; *p < 0.05; ***p < 0.001).

*HdhSPARC* showed upregulated expression according to growth pattern (stunted growth to rapid growth). Significantly higher expression (*p* < 0.05) was observed in rapid-growth abalone (Fig. 5). In contrast, the lowest expression was observed in the stunted growth abalone, but this expression level was not significantly different from that of the minimum and normal growth abalone.

### *HdhSPARC* expression during muscle remodeling and shell biomineralization of Pacific abalone

**Figure 6:**
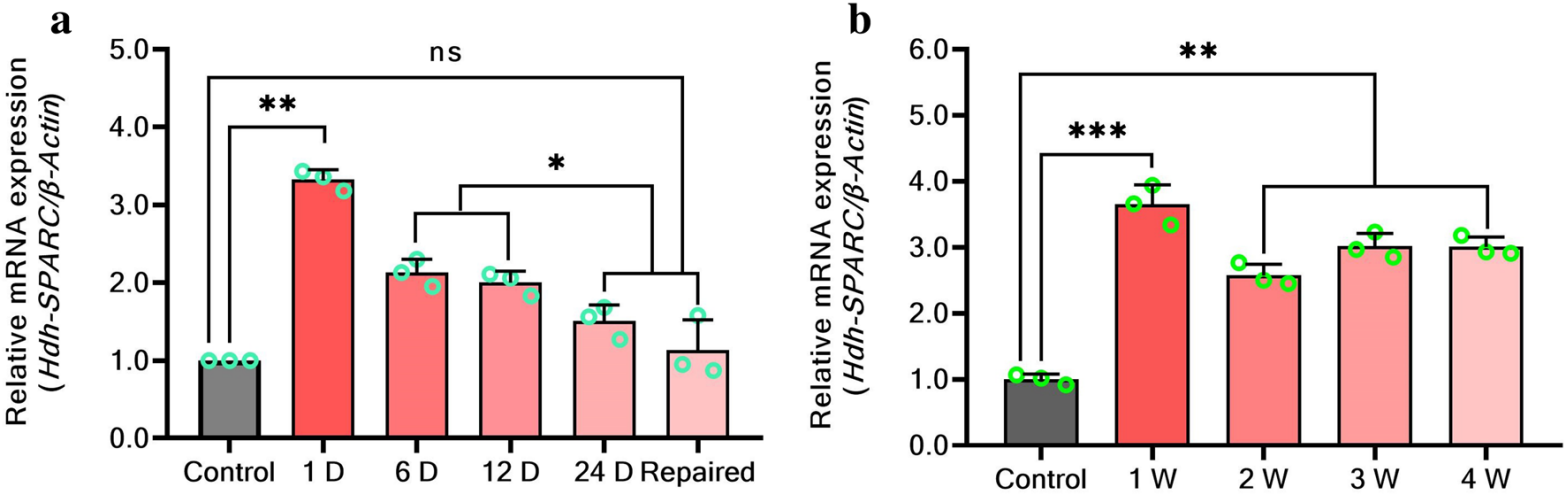
Expression of *HdhSPARC* during muscle remodeling and shell biomineralization and injured Pacific abalone. D, day; W, week. (^ns^*p* > 0.05; *p < 0.05; ***p < 0.001).

Expression analysis in muscles injured Pacific abalone showed that *HdhSPARC* was significantly (*p* < 0.05) expressed on the first day of injury (Fig. 6a). After a rapid reduction at 6 days, the expression of *HdhSPARC* decreased slowly. However, the expression was higher throughout the remodeling period than in control and remodeled abalones. The expression in the control and remodeled abalone was not significant.

Figure 6b illustrates the expression of *HdhSPARC* mRNA during the shell biomineralization of Pacific abalone. In the first week, the expression was significantly higher than that of the control abalone. Afterward, a significant decrease in expression was observed in the second week, which was increased in the remaining experimental period but was not significant. Overall, the expression of *HdhSPARC* was significantly higher than that of control abalone during the study period.

### *HdhSPARC* mRNA expression in heat-stressed and LPS exposed Pacific abalone

**Figure 7:**
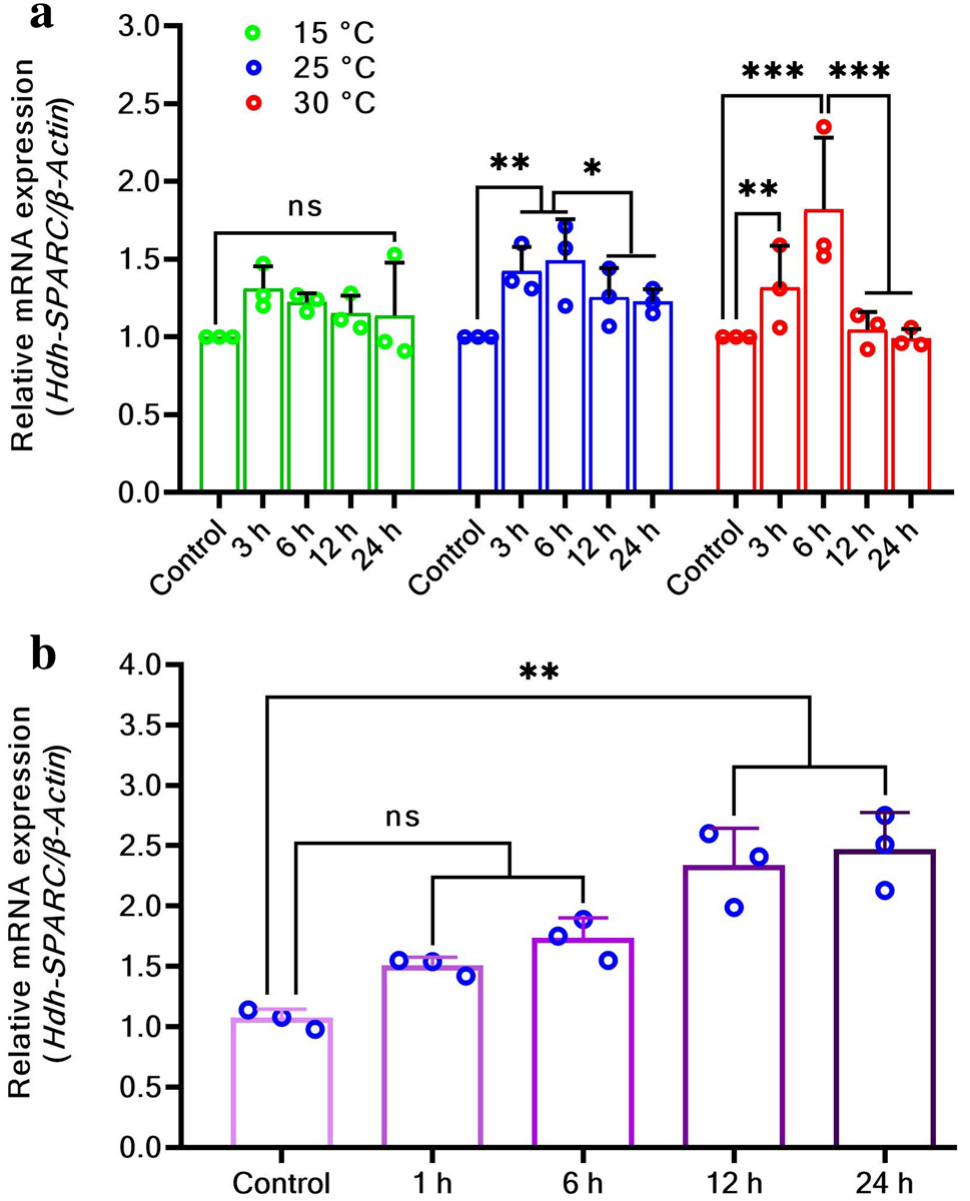
Expression of *HdhSPARC* in stressed abalone. (**a**) heat stressed, (**b**) LPS-exposed Pacific abalone. (^ns^*p* > 0.05; *p < 0.05; ***p < 0.001).

In heat stress treatment, the expression of *HdhSPARC* mRNA increased for the first 3 h. Thereafter, the expression of 15 °C treatment slowly decreased in a time-dependent manner until 24 h of treatment. However, the expression of *HdhSPARC* mRNA at 25 °C and 30 °C exhibited increasing trends until six hours of heat treatment, when it reached its peak. After that, *HdhSPARC* expression decreased significantly in the 30 °C treatment, reaching levels that were even below that of the control treatment after 24 h (Fig. 7a). On the other hand, although the expression decreased gradually in the 25 °C treatment, this effect was not significant when compared with the 30 °C treatment. Upon LPS exposure, a gradual increase in *HdhSPARC* mRNA was observed with increasing post-exposure time. The expression increased slowly for the first six hours compared to the last twelve hours, and maximum expression was observed after 24 h of LPS exposure (Fig. 7b).

### ***HdhSPARC*** mRNA expression in Cd- and H_2_O_2_-exposed Pacific abalone

**Figure 8:**
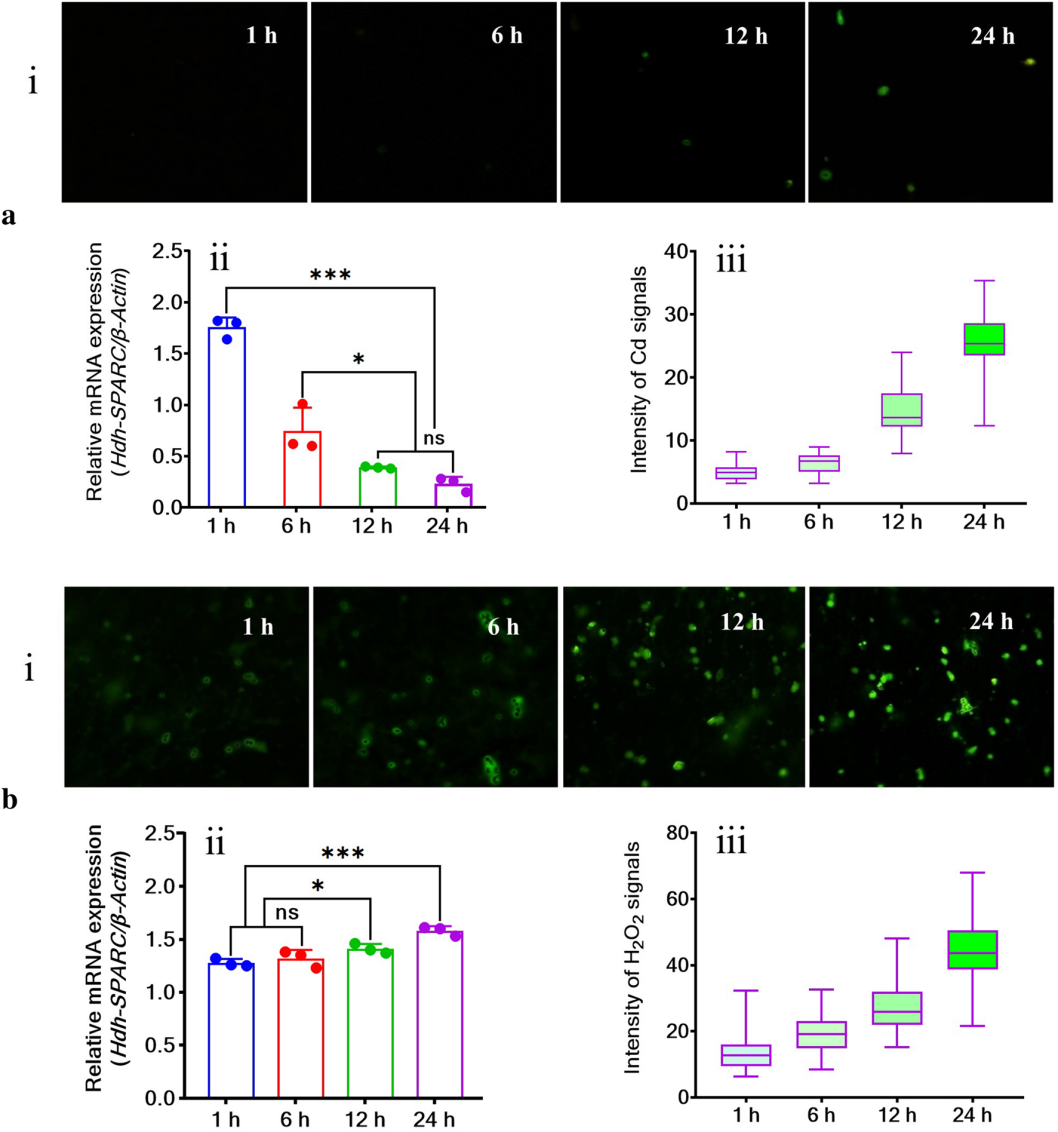
Expression of *HdhSPARC* in stressed abalone. (**a**) Cd-exposed, (**b**) H_2_O_2_-exposed Pacific abalone*.* (^ns^*p* > 0.05; *p < 0.05; ***p < 0.001).

A reverse relationship was observed between the Cd intensity and mRNA expression of *HdhSPARC* in Cd-exposed Pacific abalone. With increased post-exposure time, the intensity of the Cd signals increased (Fig. 8ai,aiii) when the expression levels decreased (Fig. 8aii). A significantly higher (*p* < 0.05) expression of *HdhSPARC* was observed after an hour of Cd exposure, whereas a lower expression level was observed at 24 h of Cd exposure. The time-dependent fluorescence signals of H_2_O_2_, mRNA expression, and fluorescence signal intensity are illustrated in Fig. 8b. *HdhSPARC* expression was found to increase with increasing post-exposure time (Fig. 8bi). The mRNA expression of *HdhSPARC* (Fig. 8bii) and intensity of the H_2_O_2_ signals (Fig. 8biii) were also significantly (*p* < 0.05) increased in a time-dependent manner, except for the first six hours, when the increase in expression was not significant.

## Discussion

Several ECM proteins are known to be inherently involved in ECM deposition, cell–matrix interaction, and growth factor signaling and play pivotal roles in regulating multiple hallmarks of ontogenetic development^10^. SPARC is a multifunctional ECM protein with a high affinity for cations and hydroxyapatite that is found in both vertebrate and invertebrate species. To gain new insights into the cellular and molecular mechanisms that govern embryogenesis and postnatal development, muscle repairment, shell biomineralization, gonadal development, and immune response under different stress conditions, the matricellular protein SPARC was cloned and its expression was analyzed in Pacific abalone.

HdhSPARC has a putative signal peptide at its N-terminal region, thus confirming its nature as a secreted protein^29^. The signal peptide produces a mature peptide of 265 amino acids. A total of 18 cysteine residues were identified in the amino acid sequence, of which 13 residues were found to be identical when compared with other invertebrate and vertebrate species.

Structurally, the HdhSPARC protein comprises three biological structural domains: a DUF domain (I) at the N-terminal region, a Kazal-type serine protease inhibitor (central domain II), and a Ca^2+^-binding extracellular domain (III), which is an alpha-helix-rich region containing a high-affinity Ca^2+^-binding EF-hand. The N-terminal domain of SPARC is highly variable in invertebrates^30^, structurally acidic, thus binding Ca^2+^ with low affinity, and rich in glutamic acid in most cases^9^. Although the first domain (domain I) of HdhSPARC is dominated by glutamic acid and aspartic acid, the conserved domain did not exhibit this glutamic acid-rich region. The central domain (domain II), a Kazal-type serine protease inhibitor (Kazal-type 1, Kazal-type 2), is cysteine-rich and contains a highly conserved N-linked glycosylation site, which is an important trait for collagen affinity and protein functionality^31^. This domain plays vital roles, including endogenous protease regulation, blood coagulation, cell development, and the immune response^32^. The third domain (domain III) is an extracellular calcium-binding domain, including an EF-hand motif with high affinity towards Ca^2+^, and has been shown to bind collagen in a Ca^2+^-dependent manner^33^.

Amino acid-based multiple sequence alignment indicated that the SPARC protein of *Haliotis discus* was highly conserved compared to that of its vertebrate and invertebrate counterparts. The HdhSPARC showed the highest identity (99.65%) with the Hdd-SPARC, thus assuming the functional similarity of the protein. The results of our phylogenetic analysis based on protein sequence indicated that SPARC was present in both vertebrate and invertebrate species^29^. HdhSPARC clustered with its molluscan SPARC, which has a close functional relationship with the arthropod clade within the vertebrate and invertebrate clusters, indicating that invertebrate SPARC is functionally different from vertebrate SPARC.

The higher expression of *HdhSPARC* in different types of tissues (testis, mantle, muscle, and digestive gland) was indicative of multifunctional characteristics. Previous studies suggest that SPARC has functions in testis development^34^, shell formation by secreting glycoprotein from the mantle^21^, muscle development and regeneration^35^, metabolism, and immune response in the digestive gland^36^. However, this expression pattern can vary depending on the development stage, health status, and environmental conditions^37^.

In this study, we analyzed the expression patterns of *HdhSPARC* in the embryonic and larval development stages of Pacific abalone and found higher expression levels at the trochophore, veliger, and juvenile stages compared to the early cell division stages. The Pacific abalone undergoes a dynamic metamorphosis during its early development stages^38^, including shell biomineralization, muscle formation, formation of the basement membrane, and embryonic hematopoiesis. Gene ontology (GO) analysis (Fig. S4) also predicted that *HdhSPARC* is involved in developing anatomical features^39^. In the trochophore and veliger stages, complex physiological and morphological processes, including shell formation, cilia development, body shape development, and head-food differentiation, occur between the transition of these two stages^40^. A previous study on *Caenorhabditis elegans* suggested that SPARC is required for the fertility and viability of embryos. Knockdown of SPARC negatively affects inner ear and cartilage development in zebrafish, indicating that SPARC is required for cartilage morphogenesis^10^. In *Patella vulgata*, *SPARC* was localized in a ring around the larval shell field, indicating its involvement in shell development^23^. However, although the involvement of *SPARC* in early embryonic and larval development has previously been studied in different species, its precise function is largely unknown.

In this study, we investigated the mRNA expression of *HdhSPARC* in different testicular development stages and found that this gene was upregulated in PS and GS but down-regulated in SS and RS, indicating that *HdhSPARC* may have potential role in germ cell maturation of male organisms. Upregulated expression of SPARC was also previously reported in mice during fetal testis development^41^. *HdhSPARC* may be involved in the transport of Ca^2+^ and Fe^2+^ cations in the testes^42^. This was further supported by the results of our GO analyses (Fig. S4), which predicted that this gene was involved in calcium ion binding. Additionally, the 3D structure of the protein (Figure S3) contained an SF4 ligand (iron/sulfur cluster) that requires Fe^2+^ ions to become active. SPARC may also be involved in the formation of sperm bundles in the testis^43^. However, this has not yet been confirmed in abalone.

The growth of Pacific abalone was significantly influenced by *HdhSPARC*. The low expression of this gene in individuals with stunted growth coupled with a significantly higher expression in rapid-growth juvenile Pacific abalone suggested that *HdhSPARC* may be involved in the growth of Pacific abalone. In mice, the level of intracellular SPARC was reported to increase with the administration of growth hormone (GH)^44^. Moreover, SPARC-deficient mice also exhibited reduced insulin secretion (a potent growth-promoting hormone)^45^, thus confirming that *HdhSPARC* may be related to the growth of Pacific abalone. SPARC regulates growth development by cell proliferation, migration, and differentiation through MEK/ERK, TAK1 and p38 signaling pathways^46^.

As a myokine, SPARC was previously predicted to participate in muscle damage repair in muscle satellite cells^12^. The present study found that the mRNA expression of *HdhSPARC* was increased during muscle regeneration in Pacific abalone, indicating that it plays a functional role in muscle tissue repair and regeneration, although the molecular mechanism is unknown yet. However, mice with null-Sparc also generate muscle, indicating that a lack of SPARC does not necessarily affect muscle development^47^. So, it can be hypothesized that presence of SPARC is compensatory but not compulsory in damage tissue repairment. Deficiency of SPARC may not affect tissue repairment pathway, other muscle development genes may be active and complete the muscle development pathway. There are lots of genes responsible for damaged tissue replacement including SPARC.

SPARC is a common biomineralization-related protein in vertebrates and mollusks and is considered a shell matrix protein due to its shell-biomineralizing functionality^48^. The expression levels of *HdhSPARC* mRNA increased after shell puncture, indicating that *HdhSPARC* may be involved in shell formation in the Pacific abalone. SPARC regulates the crystallization of CaCO_3_ through the binding of Ca^2+^ by both its amino- and carboxyl-terminal domain and induces vaterite formation in the calcite crystallization system^11^. Previously, the expression of SPARC mRNA was reported to increase in extrapallial fluid (i.e., an aqueous fluid located between the outer mantle epithelium and the inner face of the shell) in *Pinctada fucata* after shell-notching^22^, suggesting its participation in the shell repair process. The mantle is believed to be involved in prismatic layer ingrowth and thickening of the nacreous layer^49^, and *HdhSPARC* was highly expressed in the mantle during the shell biomineralization of Pacific abalone.

SPARC is a secreted heat-shock protein that is induced by heat shock and other forms of stress, with its secondary structure remaining unchanged up to 50 °C^50^. The *HdhSPARC* expression of the heat-stressed abalones was higher than that of the control abalone during the experiment, indicating that the secretion of Pacific abalone SPARC was increased. These findings were consistent with those of a previous study that analyzed SPARC expression during heat-shock treatment in chick embryos^51^. Another study revealed that the expression of SPARC was associated with other heat shock proteins such as SHP70 and HSP47^34^. Therefore, the higher expression of *HdhSPARC* during the heat shock treatment indicates its role in the protection of tissues or cells during a transient stressful state.

Furthermore, the present study observed that *HdhSPARC* is LPS-inducible and therefore LPS-exposed abalones exhibited a significantly higher *HdhSPARC* expression level compared to the control. Given that LPS is a pathogen-associated immune stimulant^52^, the higher expression of *HdhSPARC* suggests that this secreted protein also possesses immune functions. Although the specific reason for the induction of SPARC by LPS is unknown in abalone, previous studies suggest that LPS-induced SPARC is required for endothelial cell proliferation at the damage site^27^ and has been linked to changes in cell morphology and the modeling of the germinal center microenvironment^53^.

The heavy metal Cd is toxic to most aquatic animals, including marine invertebrates, and can affect growth and developmental, reproductive, hematological, and immunological functions^54^. The present study found a reverse relationship between Cd absorption and *HdhSPARC* expression in Pacific abalone. Although Cd absorption was previously reported to induce the generation of reactive oxygen species (ROS) that cause oxidative stress and eventually impair cell and tissue functions^55^, exposure of cells or tissues to high levels of Cd was also previously reported to decrease the expression level of SPARC^26^. In this study, exposure experiments were conducted using a high (6 mgL^−1^) CdCl_2_ dose. However, the specific molecular mechanism through which Cd decreases the expression level of *HdhSPARC* in Pacific abalone remains unknown.

H_2_O_2_ is an ecological factor with potential biological and chemical reactivity that can trigger oxidative stress and affects the growth, survival, and metabolic rate of aquatic organisms^56^. In this study, the expression level of *HdhSPARC* was upregulated in the digestive gland with increasing post-induction time after H_2_O_2_ injection. Fluorescence analyses detected a gradual increase of H_2_O_2_ intensity after induction, which can cause lipid peroxidation^57^. Given that the lack of SPARC increases the accumulation of reactive oxygen species, protein oxidation, lipid peroxidation, and DNA damage^58^, *HdhSPARC* may play a role in preventing lipid peroxidation in Pacific abalone.

Collectively, our findings revealed that *HdhSPARC* has a dynamic role that starts at the very beginning of Pacific abalone development. *HdhSPARC is* expressed differentially depending on body development, growth phases, reproductive period, and diverse environmental conditions, thus confirming that the variations in its expression are not only stage-specific but also organ-specific. Understanding the regulatory nature of *HdhSPARC* through expression analysis would enable the development of strategies for the rearing of abalone with slow and stunted growth to increase *HdhSPARC* expression levels, which may improve growth rates. *HdhSPARC* expression can also serve as an indicator of environmental stress and injury, thus enabling the detection of abnormal conditions and the implementation of corrective measures if necessary. Therefore, the data acquired in this study provide insights into the effects of different stressors on *HdhSPARC* expression and could thus be applied to the Pacific abalone aquaculture industry.

## Materials and methods

### Ethical statement

The present study is reported in accordance with the ARRIVE guidelines. The experimental protocols used in this study were approved by the Animal Care and Use Committee of Chonnam National University (approval number: CNU IACUC-YS-2022-8) and according to Article 14th of the Korean Animal Protection Law of the Korean government. The present study was conducted following the Guidelines for the Care and Use of Laboratory Animals of the National Institutes of Health. To minimize pain and discomfort during the sample collection from the abalones, anesthesia (5% MgCl_2_) was applied following the standard operating procedure of Chonnam National University.

### Experimental organisms and sample collection

A total of 215 reproductively mature Pacific abalone (male and female) with a mean body weight of 122.6 ± 0.59 g and a mean shell length of 88.2 ± 0.39 mm were randomly collected for different experiments from the abalone sea cage culture site at Wando-gun and transported to the Tou-Jeong Soosan abalone hatchery in Dolsan-eup, Yeosu, South Korea, where they were cultured for one month with sufficient food and water supply to recover from the transportation-related stress.

### Tissue collection for gene cloning and expression analysis

A total of two mature Pacific abalone (male and female) were sacrificed for molecular cloning purposes after anesthetizing with 5% MgCl_2_, and hemocytes, heart, testis, ovary, pleuropedal ganglion, cerebral ganglion, gill, muscle, mantle, and digestive gland were collected. Immediately after collection, tissue samples were washed with PBS (phosphate-buffered saline, 0.1 M), flash-frozen in liquid nitrogen (LN_2_), and stored at − 80 °C until total RNA extraction.

### Fertilization and sample collection during the early larval development (ELD) stages

Three-year-old reproductively mature 12 females and four male’s abalone were induced for artificial fertilization. Among them, three females and two males were responded to spawn. After spawning, artificial fertilization was conducted following the procedure described previously. After fertilization, samples of fertilized eggs, 2-cell and 4-cell stages, blastula, trochophore larvae, veliger larvae, and juveniles were collected through microscopic observation. The samples were immediately flash-frozen in LN2 and stored at − 80 °C until total RNA extraction.

### Culture of juvenile abalone and growth type sample collection

After approximately 120 h of fertilization, when they were ready for settlement, approximately 10,000 larvae were transferred to three culture tanks, each with diatoms precoated on transparent and flat plastic film (30 × 30 cm, 0.2–0.3 mm thickness). According to the size of the rearing tank, several plastic films were joined and placed at the tank bottom for convenient management. The early juvenile abalone fed on diatoms adhered to plastic films and artificial feed consisting of powder fish meal, kelp powder, micronutrients, and effective microorganisms. In South Korea, powder feed and several artificial granular feeds of different sizes are commercially available for abalone nursing at the farm level. Abalone fed with artificial feed tends to grow faster than those fed with only algae. After one year of culturing in the tank, juvenile abalone samples exhibiting different growth patterns [rapid growth, normal growth (average growth), minimal growth (growth rate less than average), and stunted growth (seems to stop growth)] were collected based on length and weight data. Muscle tissue samples were collected from each growth type of juvenile abalone, flash-frozen in LN_2_, and stored at − 80 °C until total RNA extraction.

### Tissue collection from muscle-injured tissue of Pacific abalone

A total of 18 mature Pacific abalones (three-year-old) with a mean weight of 121.4 ± 0.53 g and a mean shell length of 84.11 ± 0.51 mm were used for the muscle tissue remodeling experiment. Briefly, 3 × 1 mm of muscle tissue was carefully removed from each abalone using a tissue collecting needle after anesthetizing with 5% MgCl_2_, after which they were cultured in a rearing tank with continuous aeration, water, and food supply. Muscle tissue samples from injured abalone were collected at a one-week interval. Tissue samples from control abalone and remodeled abalone were also collected at the beginning of the experiment and after injury repair. The samples were washed with PBS immediately after collection, flash-frozen in LN_2_, and stored at − 80 °C until total RNA extraction.

### Tissue collection during shell biomineralization

Ten-month-old 20 juvenile Pacific abalones were used for the shell biomineralization experiment. The shell of each abalone was punctured (1 mm round orifice) near the mantle tissue using a perforator and then cultured in a rearing tank with continuous aeration, water, and food supply. Mantle tissues from three abalone were collected weekly. Mantle tissue from the control abalone was also collected at the beginning of the experiment. Immediately after collection, the tissues were washed with PBS, flash-frozen in liquid nitrogen, and stored at − 80 °C until total RNA extraction.

### Tissue collection of testicular developmental stages

Testis tissues were collected from five two-year-old abalones for each gonadal development stage [proliferative stage (PS), growth stage (GS), spawning stage (SS), and resting stage (RS)], and the samples were immediately flash-frozen and stored at − 80 °C until total RNA extraction.

### Tissue samples from Pacific abalones subjected to heat stress

To characterize the changes in *HdhSPARC* expression in Pacific abalones in response to stress, a heat treatment was performed at 15 °C, 25 °C, and 30 °C. For this experiment, 3-year-old Pacific abalone with a mean weight of 121.06 ± 0.41 g and a shell length of 82.8 ± 0.52 mm were collected from the sea cage abalone culture area in Wando-gun and transported to the Tou-Jeong Soosan abalone hatchery in Dolsan-eup, Yeosu, South Korea. To recover from transportation-related stress and to adjust to the hatchery environment, abalone were cultured in tanks for about 30 days with a sufficient food supply. Thereafter, 12 abalones were kept in three different aquariums for 24 h each. For the 25 and 30 °C treatment tanks, the water temperature was gradually increased (2–4 °C/h) to avoid sudden heat shock using a digital temperature controller. During this experiment, we did not provide any food for the abalone. From each treatment and control abalone (19 °C), digestive gland tissue was collected after 3, 6, 12, and 24 h, washed with PBS (0.1 M), flash-frozen in LN_2_, and stored at − 80 °C until total RNA extraction. The temperature in the control tank was 20.4 °C.

#### Lipopolysaccharide (LPS) exposures treatment

The changes in SPARC expression in response to LPS were evaluated using LPS from *Escherichia coli* O55:B5 (Sigma-Aldrich, Saint Louis, MO, USA). LPS was injected at a concentration of 10 µg/g-BW into the muscle of 16 abalone, while an equal amount of PBS was injected into the muscle of three control abalone. Digestive gland tissue from five abalones was collected, washed with PBS (0.1 M), flash-frozen in LN_2_, and stored at − 80 °C until total RNA extraction.

#### H_2_O_2_ exposure treatment

Next, an H_2_O_2_ induction experiment was performed on two-year-old Pacific abalones to examine the effects of oxidative stress on *HdhSPARC* expression in Pacific abalones. Prior to the treatment, the abalones were reared for 2 weeks in the culture tanks with continuous water supply and aeration. For the experiments, 50 μL of H_2_O_2_ solution (0.3 mg/mL) was injected into the muscle of twelve abalones, and an equal amount of PBS (0.1 M) was injected into three control abalones. Digestive gland tissue samples from injected abalones were collected at 3, 6, 12, 24, and 48 h, washed with PBS (0.1 M), flash-frozen in LN_2_, and stored at − 80 °C until total RNA extraction.

#### CdCl_2_ exposure treatment

Acclimatized 2-year-old Pacific abalones were subjected to CdCl_2_ (Sigma-Aldrich, St. Louis, MO, USA) at a concentration of 6 mgL^−1^ in an aquarium. Tissue samples from the digestive gland (*n* = 5) were collected at 1, 6, 12, and 24 h, washed with PBS (0.1 M), flash-frozen in LN_2_, and stored at − 80 °C until total RNA extraction.

#### Total RNA extraction and first-stand cDNA synthesis

Extraction of total cellular RNA from the collected tissue samples was performed using an ISOSPIN Cell and Tissue RNA Kit (Nippon Gene, Tokyo, Japan). First-strand cDNA synthesis was conducted from the extracted total RNA using oligo(dt) primers (Sigma) and a superscript III first-strand cDNA synthesis kit (Invitrogen, USA). The RACE cDNA (3′- and 5′-RACE) was synthesized from the extracted total RNA using a SMARTer® RACE 5′/3′ Kit (Takara Bio Inc., Japan). Total RNA extraction and cDNA synthesis were conducted following the manufacturer’s protocol.

## Cloning of SPARC mRNA sequence in Pacific abalone

### Cloning of partial sequence

Reverse transcription polymerase chain reaction (RT-PCR) was conducted for partial sequence cloning of HdhSPARC using muscle tissue cDNA, gene-specific forward and reverse primers, and GoTaq® DNA polymerase (Promega, Madison, WI, USA). The gene-specific primer used in the present study was designed from the known *H. discus discus* SPARC mRNA sequence (GenBank Accession No. AB600274.1), available at NCBI database. All primers used in this study are listed in Table S1 (see supplementary files). The reaction mixture for RT-PCR was prepared to a total volume of 50 μL, containing cDNA (1 μL), 20 pmol forward (HdhSPARC RT Fw) and reverse (HdhSPARC RT Rv) primers (1 μL each), GoTaq reaction buffer (colorless) (10 μL), dNTP mix (1 μL), DNA polymerase (0.25 μL), and ultra-pure water (35.75 μL). The RT-PCR thermal cycling conditions consisted of an initial denaturation step for 3 min at 95 °C, followed by 35 cycles of denaturation for 30 s at 95 °C, annealing for 30 s at 58 °C, extension for 45 s at 72 °C, and a final extension for 5 min at 72 °C. After completion, the PCR products were subjected to gel electrophoresis using 1.2% agarose, after which bands were purified using the Wizard® SV Gel and PCR Clean-Up System kit (Promega, Madison, WI, USA). Afterward, the purified DNA was ligated into the pGEM®-T Easy Vector (Promega, Madison, WI, USA) and transformed into DH5α chemically competent *E. coli* cells (Enzynomics, Korea). The positive clones were selected for plasmid DNA purification using a Hybrid-QTM Plasmid Rapidprep mini kit (GeneAll, Seoul, Korea) and sequenced at Macrogen (Seoul, Korea).

#### Cloning of RACE (5′- and 3′) sequence

The rapid amplification of cDNA ends polymerase chain reaction (RACE-PCR) was performed using the SMARTer® RACE 5′/3′ kit (Takara Bio Inc., Japan) for 5′- and 3′-RACE to obtain the full-length sequence of HdhSPARC. A set of gene-specific primers (5′- and 3′-RACE) was designed from the previously obtained partial sequence of HdhSPARC. Reaction mixtures for RACE PCR (3′- and 5′-RACE) were prepared using 2.5 μL cDNA (3′- or 5′-RACE), 1 μL RACE primers, 1 μL SeqAmp DNA polymerase, 5 μL universal primer mix (UPM), 25 μL SeqAmp buffer, and 15.5 μL ultra-pure water. 30 cycles of touchdown PCR were carried out for RACE-PCR. The thermal cycle conditions were set following the kit manufacturer's instructions. Thereafter, the PCR products were subjected to gel electrophoresis, and positive bands were purified using the NucleoSpin® Gel and PCR Clean-up Kit (Macherey–Nagel GmbH & Co. KG, Germany). The purified products were ligated into a linearized pRACE vector and transformed into Stellar-competent cells (E. coli HST08). Positive clones were purified and sequenced at Macrogen as previously described for the partial sequence. Finally, the 5′-RACE sequence, the initially cloned partial cDNA fragment, and the 3′-RACE sequence were combined and trimmed to obtain the full-length sequence.

#### Sequence analysis of cloned *H. discus hannai* SPARC (HdhSPARC)

The complete nucleotide and protein sequences of cloned HdhSPARC were analyzed using several online tools. The ORFfinder (https://www.ncbi.nlm.nih.gov/orffinder/) online tool was used to predict potential protein-encoding segments from the nucleotide sequence. The molecular weight and isoelectric point (pI) of the HdhSPARC protein were calculated using the ProtParam online tool (https://web.expasy.org/protparam/). The protein structure and gene ontology of HdhSPARC were predicted using the online protein structure prediction server I-TASSER-MTD (https://zhanggroup.org/I-TASSER-MTD/). The functional domains of the HdhSPARC protein were identified using the Motif Scan web tool (https://myhits.sib.swiss/cgi-bin/motif_scan). Conserved motifs in the SPARC protein of Pacific abalone and other species were identified using the GenomeNet (https://www.genome.jp/tools/motif/) online tool. Multiple sequence alignments based on SPARC protein sequences of abalone and other species were obtained using MEGA (version 11.0.13) and visualized using Jalview (version 2.11.1.7).

#### Phylogenetic analysis

The amino acid sequence from HdhSPARC was aligned with other SPARC protein sequences using the ClustalW online tool. A phylogenetic tree was constructed and edited using the MEGA software (version 11.0.13) with the maximum likelihood algorithm. The protein sequences used in constructing phylogenetic trees are presented in Table S2 (see supplementary files).

#### Three-dimensional modelling of HdhSPARC protein

The three-dimensional (3D) structure of the HdhSPARC protein was predicted using the previously mentioned protein structure and functional prediction program C-I-TASSER server. The 3D structure was visualized using the UCSF ChimeraX software (v. 1.2.5).

#### Quantitative real-time PCR (qRT-PCR) analysis

To quantify the relative mRNA expression of *HdhSPARC*, qRT-PCR analysis was performed in different tissues of Pacific abalone. Before qRT-PCR analysis, primer efficiency was tested to avoid experimental errors.

All qRT-PCR assays were conducted according to the 2 × qPCRBIO SyGreen Mix Lo-Rox kit (PCR Biosystems Ltd., UK) manual. Each qRT-PCR reaction mixture was prepared in a total volume of 20 μL, containing cDNA template (1 μL), 10 pmol gene-specific forward and reverse primers (1 μL each), SyGreen Mix (10 μL), and double distilled water (10 μL). Triplicate reactions were performed for target and reference genes in each tissue sample. The PCR amplification conditions consisted of a preincubation step at 95 °C for 2 min, followed by 40 cycles of denaturation, annealing, and extension at 95 °C for 30 s, 60 °C for 20 s, and 72 °C for 30 s. The melting temperature was set as the instrument's default setting. At the end of each cycle, a fluorescence reading was recorded for quantification. A LightCycler® 96 System (Roche, Germany) was used for amplification and data analysis. The relative gene expression was determined using the 2^−ΔΔCT^ method with the Pacific abalone β-actin gene (accession no. MW387000) as an internal reference. All primers used in qRT-PCR analysis are summarized in Table S1 (see supplementary files).

#### Fluorescence-based detection of intracellular H_2_O_2_ and Cd

The intracellular hydrogen peroxide and cadmium levels in digestive gland tissue samples were detected using an intracellular hydrogen peroxide assay kit (MAK164, Sigma-Aldrich) and Leadmium™ Green AM dye (Invitrogen, Thermo Fisher Scientific), with slight protocol modifications as described previously^59^. Sample preparation and intensity measurement were performed as described by Hossen et al.^60^. Briefly, samples were stained with a fluorescent peroxide sensor and incubated for 15 min at 37 °C in the dark. The stained cells were visualized under a fluorescence microscope (green filter: 450–490 nm, Eclipse E600, Nikon, Tokyo, Japan). The fluorescence intensity of a minimum of 100 cells was measured using ImageJ software version 1.8.0_172.

#### Statistical analysis

The mRNA expression values were statistically analyzed and expressed as the mean ± standard error. Changes in relative mRNA expression were calculated via nonparametric ANOVA analysis using the GraphPad Prism software (version 9.3.1). Statistical significance was set at a *p*-value less than 0.05. All graphs were generated using Microsoft Excel and GraphPad Prism 9.3.1 software.

### Supplementary Information

Supplementary Information.

## Data Availability

All data generated in this study is included in this article. The nucleotide and protein sequence of SPARC is available at https://www.ncbi.nlm.nih.gov/nuccore/OM937904.1. The nucleotide and protein sequences used in the present study were collected from NCBI (https://www.ncbi.nlm.nih.gov/) and are presented in Table S2.
