# Supplementary material for: Cloning, characterization, and spatio-temporal expression patterns of HdhSPARC and its responses to multiple stressors
Source: Sci Rep. 2024 Jan 26;14:2224. doi: 10.1038/s41598-024-51950-7 (PMC10817941; doi:10.1038/s41598-024-51950-7)
Supplement: Supplementary file 1 — Supplementary Information. [file 41598_2024_51950_MOESM1_ESM.pdf]

Cloning, characterization, and spatio-temporal expression patterns of *HdhSPARC* and its responses to multiple stressors

Md Abu Hanif[1], Shaharior Hossen[1], Cheol Young Choi[2] and Kang Hee Kho[1*]

Table S1. List of different primers used for cDNA synthesis, cloning, and expression analysis in this study.

Primer name	Nucleotide sequences	Purpose
**Oligo dT (OdT)**	GGCCACGCGTCGACTAGTACTTTTTTTTTTTTTTTTT	cDNA synthesis
**Oligo dT adapter (AP)**	GGCCACGCGTCGACTAGTAC	
**SPARC-Fw**	GAGCATACCTCAGACGAAG	Fragment PCR
**SPARC-Rv**	GGTCAGTTAGGACTCTAGTG	
**SPARC-5′**	GATTACGCCAAGCTTCCAGACCTACGAAAGCGAGTGTGAGC	5′ RACE PCR
**SPARC-3′**	GATTACGCCAAGCTTCACACTTACAGAATGGGGAGCCTGCC	3′ RACE PCR
**SPARC-qFw**	GCTATGGAAGTTCTGTGACC	qRT-PCR
**SPARC-qRv**	GTGTGATCTTGCGATTGCTG	
**Hdh-β-Actin-Fw**	GATAGTGCGAGACATCAAGG	
**Hdh-β-Actin-Rv**	GAGCTCGAAACCTCTCATTG	

Table S2. Sequence information of SPARC used for multiple sequence alignment and phylogenetic analysis.

Common Name	Scientific Name	Gene	GenBank Accession No.	
			Nucleotide	Protein
**Japanese disc abalone**	*Haliotis discus discus*	SPARC	AB600274	BAK22657
**Pacific abalone**	*Haliotis discus hannai*	SPARC	OM937904	UTD53616
**Akoya pearl oyster**	*Pinctada fucata*	SPARC	KU310669	AND99565
**Common limpet**	*Patella vulgata*	SPARC	HE962382	CCJ09602
**Atlantic salmon**	*Salmo salar*	SPARC	BT043718	ACH70833
**Western clawed frog**	*Xenopus tropicalis*	SPARC	AY575077	AAT01218
**Brown rat**	*Rattus norvegicus*	SPARC	NP_036788	NM_012656
**Modern humans**	*Homo sapiens*	SPARC	J03040	AAA60570
**Sumatran orangutan**	*Pongo abelii*	SPARC	NC_071990	NP_001127042
**Cattle**	*Bos taurus*	SPARC	BT030548	ABQ12988
**Wild boar**	*Sus scrofa*	SPARC	AY963262	AAX83050
**Orca**	*Orcinus orca*	SPARC	XM_004280325	XP_004280373
**Iberian ribbed newt**	*Pleurodeles waltl*	SPARC	MH206592	AWQ28573
**Rhinatrema**	*Rhinatrema bivittatum*	SPARC	MH206597	AWQ28578
**Cayenne caecilian**	*Typhlonectes compressicauda*	SPARC	MH206596	AWQ28577
**African clawed frog**	*Xenopus laevis*	SPARC	X62483	CAA44350
**Western clawed frog**	*Xenopus tropicalis*	SPARC	AY575077	AAT01218
**Japanese rice fish**	*Oryzias latipes*	SPARC	AY575076	AAT01217
**Turbot**	*Scophthalmus maximus*	SPARC	KF192603	AGW25370
**Qrange-spotted grouper**	*Epinephelus coioides*	SPARC	EU882839	ACJ66296
**Rainbow trout**	*Oncorhynchus mykiss*	SPARC	U25721	AAC99813
**Akoya pearl oyster**	*Pinctada fucata*	SPARC	AB600273	BAK22656
**Blue mussel**	*Mytilus edulis*	SPARC	CAJPWZ010002740	CAG2244558
**Mexican Freshwater Shrimp**	*Hyalella azteca*	SPARC	XM_018167385	XP_018022874
**San Francisco brine shrimp**	*Artemia franciscana*	SPARC	AB052961	BAB20042
**Water flea**	*Daphnia magna*	SPARC	XM_045173162	XP_045029097
**German cockroach**	*Blattella germanica*	SPARC	LT220510	CZQ50751
**Brown planthopper**	*Nilaparvata lugens*	SPARC	MZ983402	UPH52990

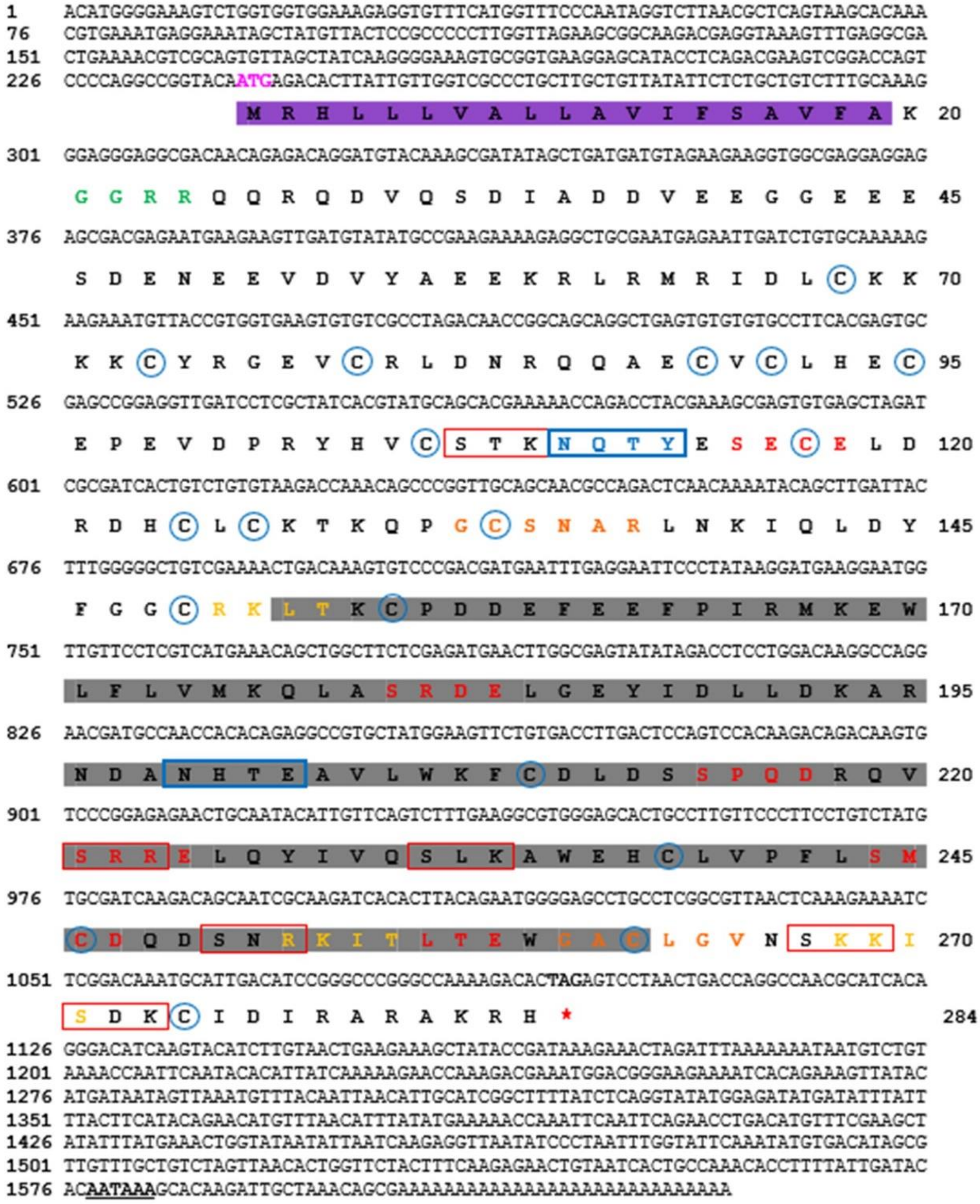

Fig. S1. Full-length nucleotide and deduced amino acid sequences of Hdh-SPARC (GenBank accession No. OM937904.1). The numbers at the left and right sides of the sequence indicate the nucleotide and amino acid positions, respectively. The start and stop codons are indicated in pink bold font and with an asterisk, respectively. The signal peptide is indicated in violet. The SPARC domain is shaded in gray. The cAMP and cGMP-dependent protein kinase phosphorylation sites are indicated in yellow font. The red boxes indicate the PKC phosphorylation sites. Predicted

casein kinase II phosphorylation sites are indicated in red. A single amidation site present in the sequence is marked in green. The blue boxes indicate the two N-glycosylation. The N-myristoylation sites are indicated in orange. The underlined bases are the putative polyadenylation signal. The blue circles indicate the conserved cysteine residues.

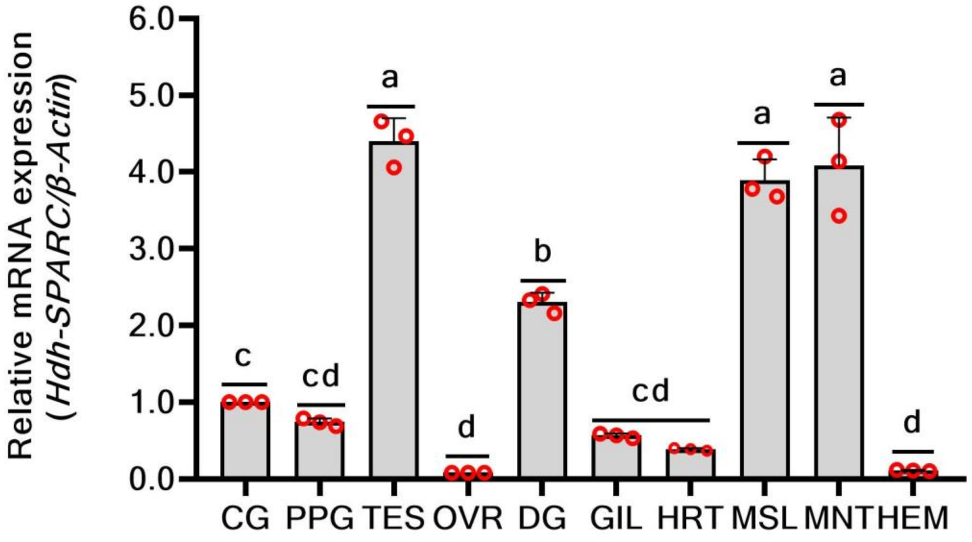

Fig. S2. *Hdh-SPARC* mRNA expression level in different tissues of Pacific abalone *H. discus hannai*. CG, cerebral ganglion; PPG, pleuropedal ganglion; TES, testis; OVR, ovary; DG, digestive gland, GIL, gill; HRT, heart; MSL, muscle; MNT, mantle; HEM, hemocyte

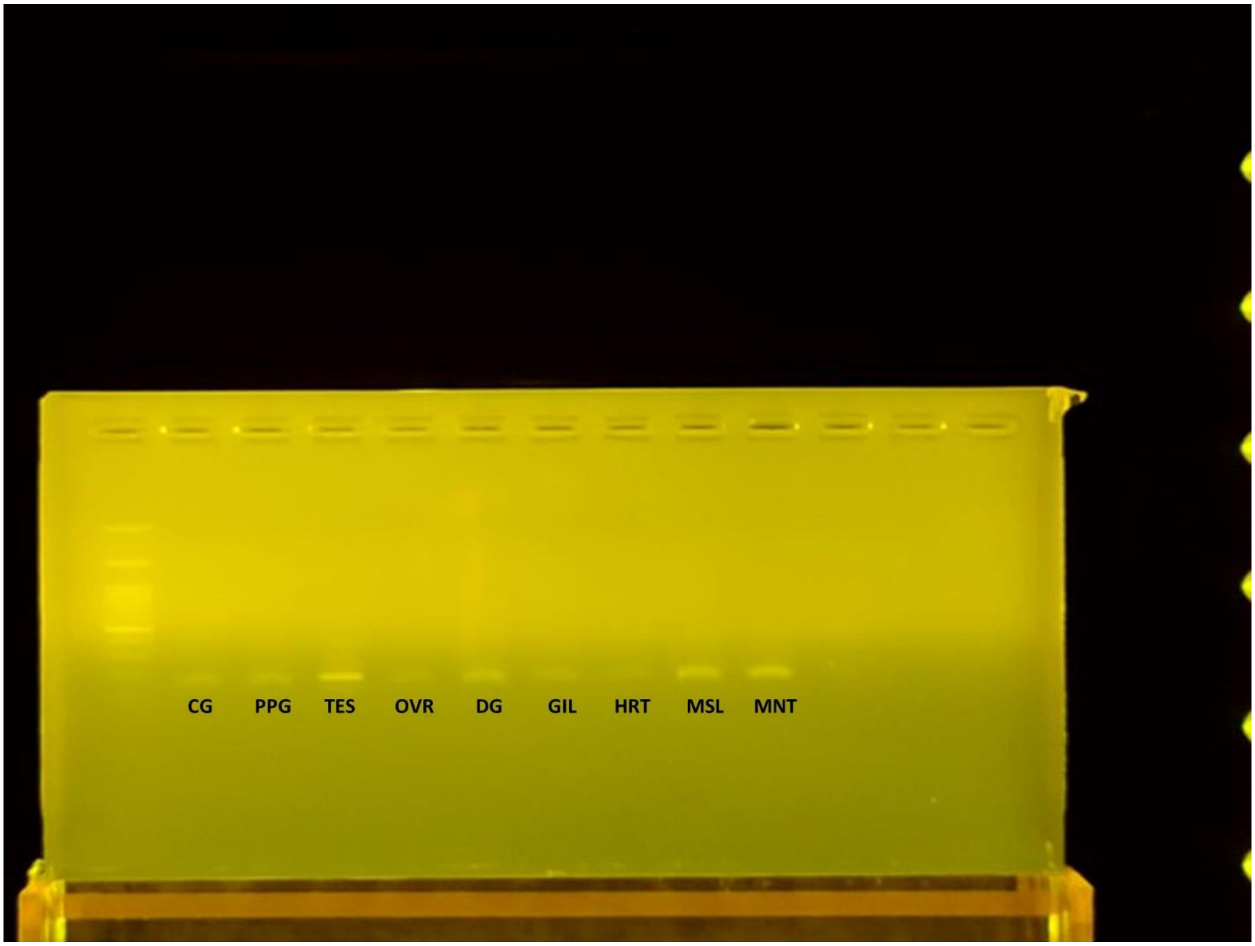

Fig. S3. Tissue specific expression of Hdh-SPARC in semi-quantitative real-time polymerase chain reaction

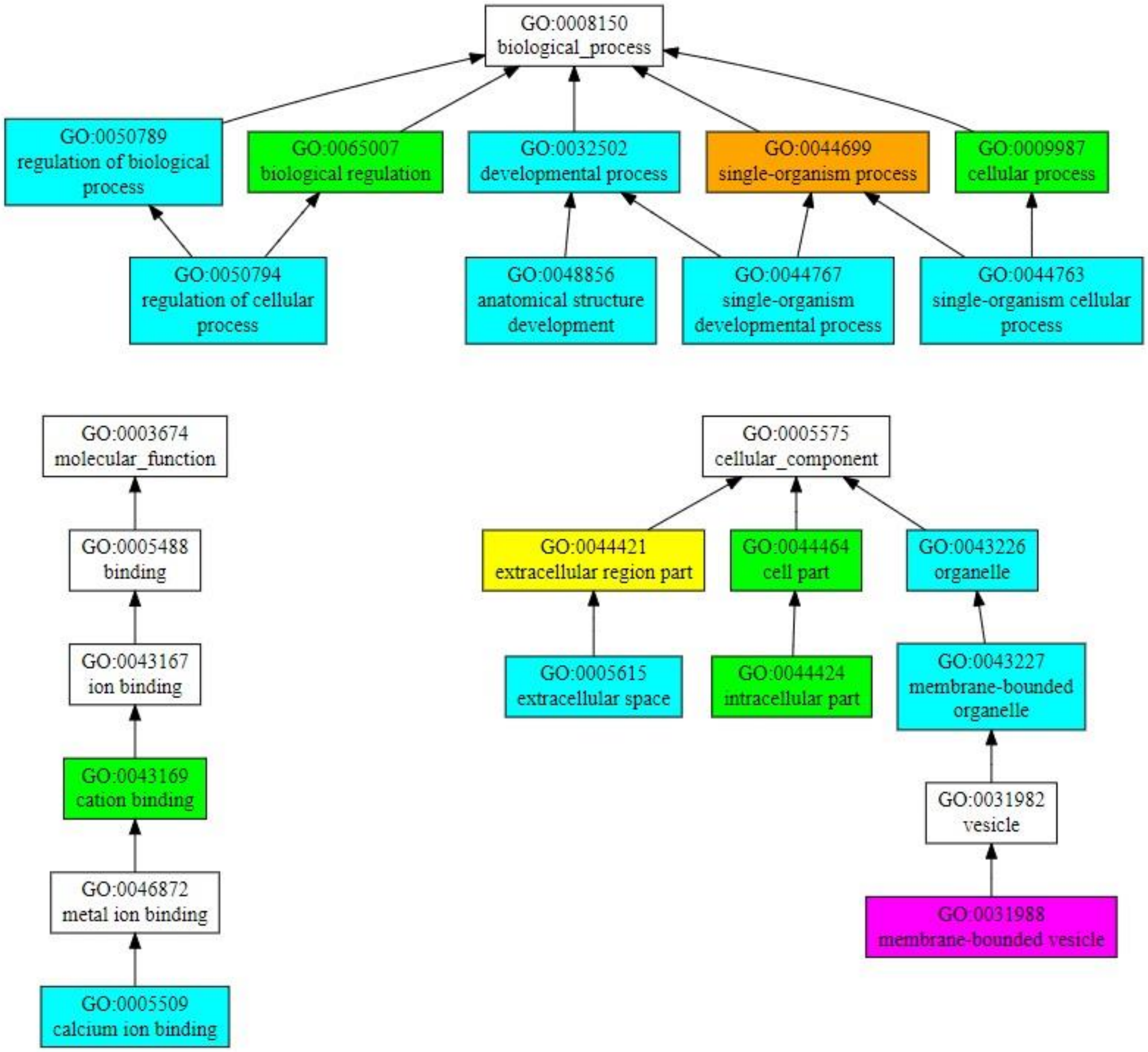

Fig. S4. Gene ontology analysis: Biological process, Molecular function, and cellular component
